# Vitamins D: Relationship between Structure and Biological Activity

**DOI:** 10.3390/ijms19072119

**Published:** 2018-07-20

**Authors:** Andrzej Kutner, Geoffrey Brown

**Affiliations:** 1 Pharmaceutical Research Institute, 8 Rydygiera, Warsaw 01-793, Poland; a.kutner@ifarm.eu; 2 Institute of Clinical Sciences, Institute of Immunology and Immunotherapy, College of Medical and Dental Sciences, University of Birmingham, Edgbaston, Birmingham B15 2TT, UK

**Keywords:** cell differentiation, vitamin D, vitamin D receptor, vitamin D analogues, crystallography

## Abstract

The most active metabolite of vitamin D is 1α,25-dihydroxyvitamin D_3_, which is a central regulator of mineral homeostasis: excessive administration leads to hypercalcemia. Additionally, 1α,25-dihydroxyvitamin D_3_ is important to decision-making by cells, driving many cell types to growth arrest, differentiate and undergo apoptosis. 1α,25-Dihydroxyvitamin D_3_ regulates gene transcription by binding to a single known receptor, the vitamin D receptor. Rapid intracellular signals are also elicited in vitro by 1α,25-dihydroxyvitamin D_3_ that are independent of transcription. There are many aspects of the multiple actions of 1α,25-dihydroxyvitamin D_3_ that we do not fully understand. These include how a single receptor and provoked rapid events relate to the different actions of 1α,25-dihydroxyvitamin D_3_, its calcemic action per se, and whether a large number of genes are activated directly, via the vitamin D receptor, or indirectly. A strategy to resolving these issues has been to generate synthetic analogues of 1α,25-dihydroxyvitamin D_3_: Some of these separate the anti-proliferative and calcemic actions of the parent hormone. Crystallography is important to understanding how differences between 1α,25-dihydroxyvitamin D_3_- and analogue-provoked structural changes to the vitamin D receptor may underlie their different activity profiles. Current crystallographic resolution has not revealed such information. Studies of our new analogues have revealed the importance of the A-ring adopting the chair β-conformation upon interaction with the vitamin D receptor to receptor-affinity and biological activity. Vitamin D analogues are useful probes to providing a better understanding of the physiology of vitamin D.

## 1. Introduction

**Figure 1:**
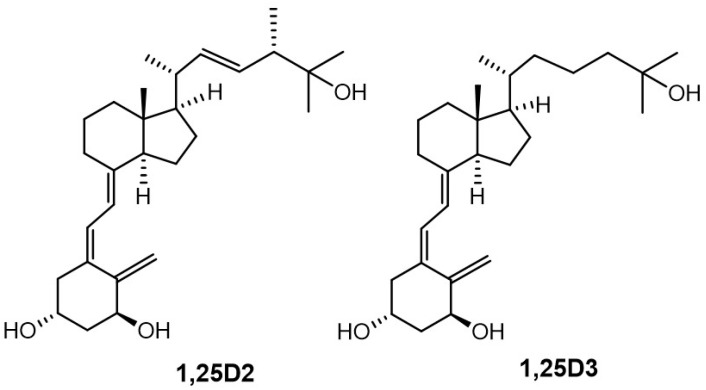
The structures of 1α,25-dihydroxyvitamin D_3_ (1,25D3) and 1α,25-dihydroxyvitamin D_2_ (1,25D2).

1α,25-Dihydroxyvitamin D_3_ (1,25D3, calcitriol, Figure 1) is the most active metabolite of vitamin D (vitD, cholecalciferol). This *seco*-steroid hormone has many biological roles [1]. A key physiological role of 1,25D3 is to regulate the absorption and transportation of essential minerals, particularly calcium, phosphorus, and magnesium, which are important to the maintenance of bone [2,3]. 1,25D3 also plays a key role in decision-making by cells because it can elicit events that result in many types of cells arresting their growth, differentiating, and undergoing apoptosis. The anti-proliferative action of 1,25D3 extends to malignant cells, leading to interest in its use for differentiation therapy of leukemia and other cancers. However, a substantial limitation to this use of 1,25D3 is that its calcemic action restricts achieving an effective therapeutic dose [4]. Many of the immune cell types express the receptor for vitamin D (VDR): 1,25D3 has an important role in their function and is, therefore, important to good health [5,6]. A related interest is the therapeutic use of 1,25D3 as an anti-inflammatory agent.

## 2. The Modes of Action of Vitamin D

Some of the biological effects of 1,25D3 result from the direct activation of target genes [7,8]. In this case, 1,25D3 binds to its single known receptor VDR. The VDR forms a heterodimer with the retinoid X receptor to regulate gene transcription. 1,25D3 regulates a large number of genes, including ones that link to the growth and differentiation of cells and diseases that include cancer, diabetes, and arthritis. At least 229 genes are subject to regulation by 1,25D3, as revealed by ChiP-seq analysis [9,10]. We do not understand whether the VDR activates all of the envisaged number of genes directly or indirectly.

For some cells, 1,25D3 treatment leads to the activation of non-genomic signaling pathways. The delineation of these events is from in vitro studies, and they occur within seconds or minutes after treating cells with 1,25D3 [11,12]. In this scenario, 1,25D3 initiates signals at the plasma membrane or in the cytosol. The mitogen-activated protein kinases and phosphatidylinositol 3-kinase signaling cascades mediate this rapid response to some extent [13,14,15]. There are putative receptors for 1,25D3 at the cell membrane that include the membrane-associated rapid response steroid-binding protein (MARRS, ERp57, or PDIA3), megalin, and cubilin, and the latter two transport vitamin D, complexed to its serum binding protein [16]. Additionally, 1,25D3 binds to the VDR in an alternative VDR-binding pocket through a 6-*cis*-1,25D conformation as proposed by Mizwicki and Norman [17]. Endocytosis of this and other putative membrane receptors can generate immediate intracellular signals *via* the Rab5/PI3-kinase pathway. A shift in the balance between VDR-provoked gene transcription and rapid signaling events might underlie the anti-proliferative versus calcemic actions of 1,25D3. However, the structure-calcemic activity relationship for most of the known vitamin D analogues is not clear to date.

## 3. Vitamin D Analogues

Over recent years, investigators have generated and studied hundreds of vitD analogues and several metabolites. Their structures are important to biological activity. A total of 17 crystal structures of 1α-hydroxylated vitDs are at the Cambridge Structural Database, and there are structures of 63 vitD analogues bound to the engineered VDR. Despite all of this, we still do not understand the molecular events that force an analogue to adopt the A-ring slightly distorted chair β-conformation *per se* and when bound to the VDR. It remains a mystery as to why the three hydroxyls (1, 3, and 25) that mediate analogue binding to the VDR are almost overlapping and why analogues have very different structures and activities.

The most important parts of vitD and analogues regarding the affinity for the VDR, and consequently activity profile, are the A-ring, the side-chain, and the CD-ring system.

Recently, we divided double point modified (DPM) analogues of vitDs [18] into structural groups. We introduced the new classification system for *semi*-quantifying the biological activities of DPM analogues and outlined new directions for structural modifications. Based on this overview, we designed new analogues of 1α,25-dihydroxyvitamin D_2_ (Figure 1, 1,25D2) to study the active conformation of the A-ring. We used a panel of closely related analogues of 1,25D2, [19] with solved crystal structures [20], rather than just a single analogue. For reference, we used a new compound (not shown) with all the functional groups (C-25 carboxy and 1- and 3-hydroxyl) protected and, therefore, deprived of electrostatic interactions [20]. The correlation of the structures of the new analogues with their biological activities [21,22] allowed us to solve a long-lasting enigma [23,24] surrounding the 1α-hydroxylated analogues of vitD. We have proposed a new general rule (see below), confirmed by theoretical calculations, which applies to all of our crystallographic data and which has been obtained for 1α -hydroxylated analogues of vitD over the last 25 years.

We investigated the side-chain structure by generating (24*Z*) geometric isomers [25] of our hypocalcemic analogues of 1,25D2. We examined their binding affinities for the VDR, the potency regarding inhibition of cell growth, and the regulation of stem cell-related gene expression in colon cancer cells [26]. Resistance to *hCYP24A1*-metabolism, which deactivates 1,25D3 by enzymatic hydroxylation [25], relates to a preferred side-chain geometry. An extended and rigidified side-chain of 1,25D2 is responsible for the longer-term biological effects of vitD (see below). We used human AML cell lines to examine the correlation between the side-chain geometry of 1,25D2 and potency in arresting cell growth and inducing cell differentiation [27].

**Figure 2:**
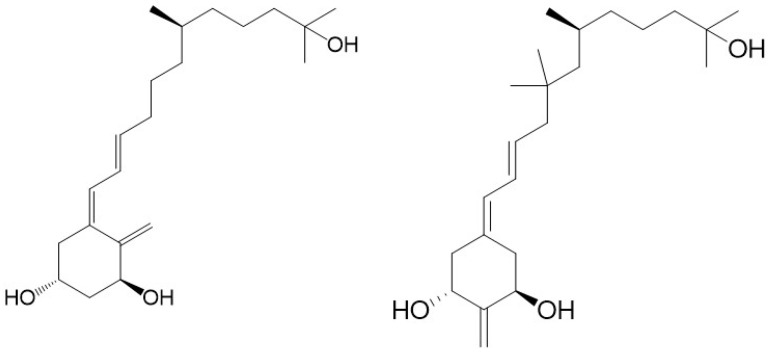
The structure of RAD_2_, the first *des*-C,D analogue of 1α,25-dihydroxyvitamin D_3_ and of (20*S*)-13,13-dimethyl-*des*-C,D-1,25-dihydroxy-2-methylene-19-*nor*-vitamin D_3_.

The original principle of CD-ring modified analogues [28] was that the entire CD-ring system of an analogue was not required for activity [29]. The hydrindane CD-ring moiety originates from the biotransformation of sterols into 9,10-*seco*-steroids (vitD), and it was not specifically biosynthesized for vitD. Additionally, the CD-ring is the only part of the vitD molecule that does not participate in the metabolic transformations of vitD. Moreover, molecular modeling revealed that *des*-C,D analogues relate structurally to the biotransformation product [29] of all-*trans*-retinoic acid (ATRA), a potent differentiating agent. *Des*-C,D analogues combine aspects of both the vitD and retinoid structures, and we call them retiferols. Our original concept of retiferols led to the synthesis of a substantial variety of CD-ring modified analogues, including other *des*-C,D analogues [30], *des*-D,C-ring analogues [31], and *des*-C,D-ring homo analogues [32], and their biological activity was evaluated [30,31,32]. Our first retiferol (Figure 2) RAD_2_ became a synthetic target [33]. Roche researchers [30] obtained 19-*nor* RAD_2_ and reported that this analogue was useful in the treatment of hyper-proliferative skin diseases in vivo. Here, we discuss how CD-ring modifications affect activity.

Below, we examine the use of various classes of new analogues of vitD to unravel some of the issues relating to structure and biological activity. Of particular importance is that new analogues separate the anti-proliferative and calcemic actions of the parent hormone more effectively than previous analogues (see below), and how might this be the case?

## 4. The Vitamin D A-Ring Conformation

**Figure 3:**
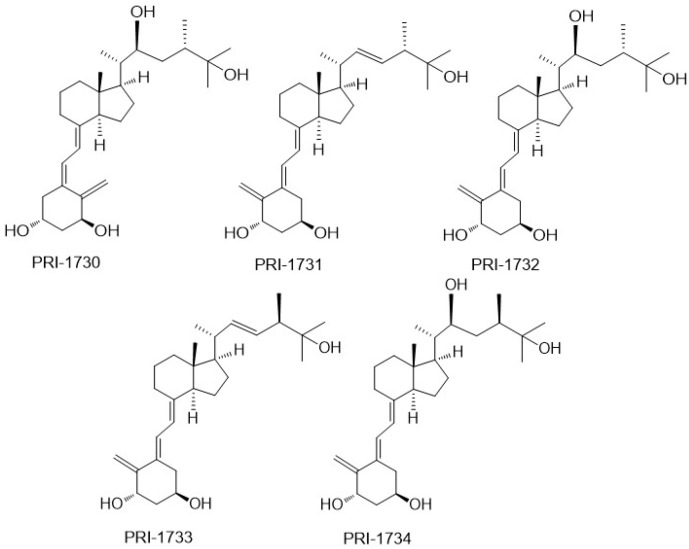
The structures of double point modified analogues of 1α,25-dihydroxyvitamin D_2_.

To investigate the correlation between the A-ring conformation and activity, we conceived and synthesized, by a novel convergent strategy, a panel of DPM analogues of 1,25D2 coded PRI-1730, PRI-1731, PRI-1732, PRI-1733, and PRI-1734 (Figure 3) [19]. Our modifications included new 5,6-*trans* (5*E*,7*E*) geometry of the A-ring *per se* or combined with the further modifications in the side chain. These included an additional (22*S*)-hydroxyl, 22,23-single bond, and a reversed absolute configuration (24-*epi*) at C-24. All our analogues induced differentiation of the VDR positive A375 and VDR negative SK-MEL 188b human malignant melanoma cell lines [22]. As expected, 5,6-*trans* modification of the A-ring was advantageous to enhancing the anti-proliferative activity of the analogues but not as a single point modification. Very unexpectedly, the additional 22-hydroxyl in the side-chain, conceived to enhance VDR binding, reduced significantly the anti-proliferative activity of both the natural and 5,6-*trans* series of analogues [21].

PRI-1731 and PRI-1733 increased translocation of the VDR to the nucleus of HL60 cells but to a lesser extent than provoked by 1,25D2 and 1,25D3. 5,6-*Trans* modification contributed substantially to the increased stability of the PRI-1731 and PRI-1733 against enzymatic hydroxylation by *h*CYP24A1, produced by expressing recombinant protein in *Escherichia coli*. Unexpectedly, reversing the chirality at C-24 from the natural (24*S*) in PRI-1731 to the (24*R*) in PRI-1733 did not affect metabolic resistance. The conversion remained at the high level of only 12% for both analogues, compared with 44% and 35% for 1,25D3 and 1,25D2, respectively. The addition of 22-hydroxyl and the saturation of the 22,23-double bond resulted in a dramatic loss of metabolic stability from 12% for PRI-1731 to a 52% conversion for PRI-1732. We used fluorescence polarization-based competition assay to measure the binding affinity of our analogues for the VDR. Only the 5,6-*trans* analogue of 1,25D2 (PRI-1731) showed a binding affinity comparable to that of both 1,25D2 and 1,25D3. Very intriguingly, a combination of all four structural modifications resulted in a complete loss of activity in the case of PRI-1734. This analogue showed weak binding to the VDR [21] and failed to agonize the VDR. However, its structure might be a good starting point for the design of a vitD antagonist, once the binding is improved [21]. The modifications introduced have not led to an increase in differentiation-inducing potency for the above new panel of analogues. However, they have resulted in a very divergent group of analogues that have provided very important data regarding structure versus activity relationships.

VitD analogues are resistant to crystallization due to a high flexibility over the number of rotated single bonds in the side-chain and in the triene system. Therefore, we were very fortunate to obtain single crystals of as many as three analogues (PRI-1730, PRI-1731, and PRI-1732), out of a panel of our five analogues [19], suitable for X-ray diffraction. For our structure–activity relationship, it was also of key importance to obtain a single crystal of the synthetic intermediate with all the functional groups (1,3, and 25-hydroxyl and 25-carboxyl) protected and, therefore, deprived of electrostatic interactions [25]. Very interestingly, we observed that the A-ring of PRI-1730 and PRI-1731 exists in a crystal state in a chair β-conformation, and that of PRI-1732 and of the totally protected synthetic intermediate in a chair α-conformation [20]. Using this unusually large collection of solid-state structures, our crystallography study revealed the new general rule regarding the solid-state A-ring conformation. We concluded that the direct hydrogen bond between 1-hydroxyl and 3-hydroxyl of the neighboring molecule forces the A-ring of 1α-hydroxylated analogues of vitamin D to adopt the chair β-conformation. According to our rule, the analogues adopting an A-ring chair β-conformation exhibit higher biological activity than the structurally related analogues existing in the chair α-conformation. This is due to the possibility of stronger electrostatic interactions of the chair β-conformation with the VDR. This explains why PRI-1730, which adopts the chair β-conformation, shows much higher activity (e.g., in nuclear translocation of the VDR) than PRI-1732, which has the chair α-conformation, and why PRI-1730 shows a much higher metabolic stability (31% conversion) than PRI-1732 (52% conversion). Contrary to the common understanding, our crystallographic studies demonstrated that the structure of an analogue in a solid state very much relates to its structure in a solution and when interacting with the VDR, predicts its biological activity as high or low. We should consider testing only the analogues existing in a solid state in A-ring chair β-conformation for biological activity.

## 5. Modifications to the Vitamin D Side-Chain

**Figure 4:**
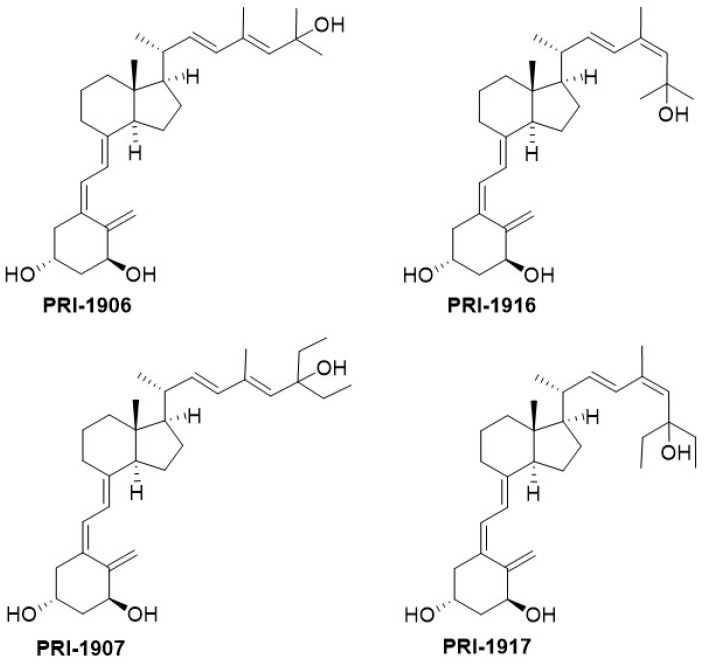
The side-chain extended and rigidified analogues of 1α,25-dihydroxyvitamin D_2_ (PRI-1906 and PRI-1907) and their (24*Z*) geometric isomers (PRI-1916 and PRI-1917).

In our studies of the relationship between the side-chain structure and activity, we developed another new convergent strategy to modify our leading 1,25D2 analogues, PRI-1906 and PRI-1907 (Figure 4). We extended and rigidified the side-chain and obtained new analogues, PRI-1916 and PRI-1917, with the previously unknown geometry at C-24 [(24*Z*), instead of (24*E*)]. The binding affinity of PRI-1916, with two methyl at the terminus of the side-chain at C-25, for the full-length human VDR in a fluorescence polarization assay was substantially higher than that of the previously obtained PRI-1906. However, the affinity of PRI-1917, with two ethyl at C-25, was much lower than that of the parent PRI-1907. This finding indicated that terminal alkyls at C-25 strongly influence the binding affinity of analogues for the VDR. Our PRI-1906 and PRI-1907 have a very high resistance to metabolic conversion (2.3% and 0.8% for PRI-1906 and PRI-1907, respectively, compared with 44% and 35% for 1,25D3 and 1,25D2, respectively). Our new PRI-1916 and PRI-1917 showed a somewhat lower resistance to conversion, although still higher than that of 1,25D3 and 1,25D2. From these findings we proved that a rigid and straight (24*E*) geometry of the side-chain is preferred for metabolic resistance, and in keeping (24*E*), (24-*trans*) analogues elicit long-term biological effects against cancer cells [25]. (24*Z*) Modification of the side-chain of 1,25D2 analogues has a contrasting effect on the differentiating activity of PRI-1906 and PRI-1907. Although the VDR affinity of the (24*Z*) analogue PRI-1916 was lower than that of (24*E*) analogue PRI-1906, the potency of PRI-1916 was slightly higher than that of PRI-1906 when tested against the human AML cell lines KG-1a, HL60, U937, and MOLM-13, which typify different stages of myeloid maturation [27] However, PRI-1917 was significantly less potent than PRI-1907. We, therefore, finally concluded that the (24*E*) side-chain geometry combined with selected modifications of the A-ring is preferable in terms of generating potent anti-proliferative and differentiating vitDs. Evaluation of the differentiating activity and calcemic action of newer analogues of 1,25D2 has shown they are more potent differentiating agents than 1,25D3 and have a reduced calcemic action (see Table 1).

The values obtained for 1α,25-dihydroxyvitamin D_3_ (1,24D3) are shown for comparison. The EC_50_ for differentiating potency is the value that drives half-maximal differentiation of the promyeloid cell line HL60 towards neutrophils. Calcium levels are the mean of the values obtained for five mice treated with 0.3 µg/kg of 1,25D3 or an analogue every other day for 3 weeks and measured on day 21. The serum calcium level in ethanol-treated mice was 62 µM. The analogues PRI-1907, PRI-5201, and PRI-5202 are more potent differentiating agents than 1,25D3 and have a lower calcemic action.

Quite unexpectedly, both (24*E*) and the new (24*Z*) analogues are equipotent in decreasing the cloning capacity and the proliferative activity of the human colon cancer cells HT-29 [26]. These cells are refractory to the anti-proliferative action of the chemotherapeutic agent 5-flurouracil. Both of the C-25 diethyl analogues, PRI-1907 and PRI-1917, decreased the level of expression of stemness-related genes, while both dimethyl analogues, PRI-1906 and PRI-1916, were not able to downregulate these genes. Therefore, the new geometric analogues, PRI-1907 and PRI-1917, are good candidates for further studies to examine the benefit to preventing cancer relapse. In this regard, they act to decrease the proliferative capacity of cells that initiate tumor regrowth, by virtue of downregulating stemness-related genes. The analogues might be useful in post-treatment of cancer patients with conventional reductive chemotherapy, particularly regarding the reduced calcemic action of PRI-1907 and the newer analogues of 1,25D2 (see Table 1) [26].

## 6. CD-Ring Modifications of Vitamin D

**Figure 5:**
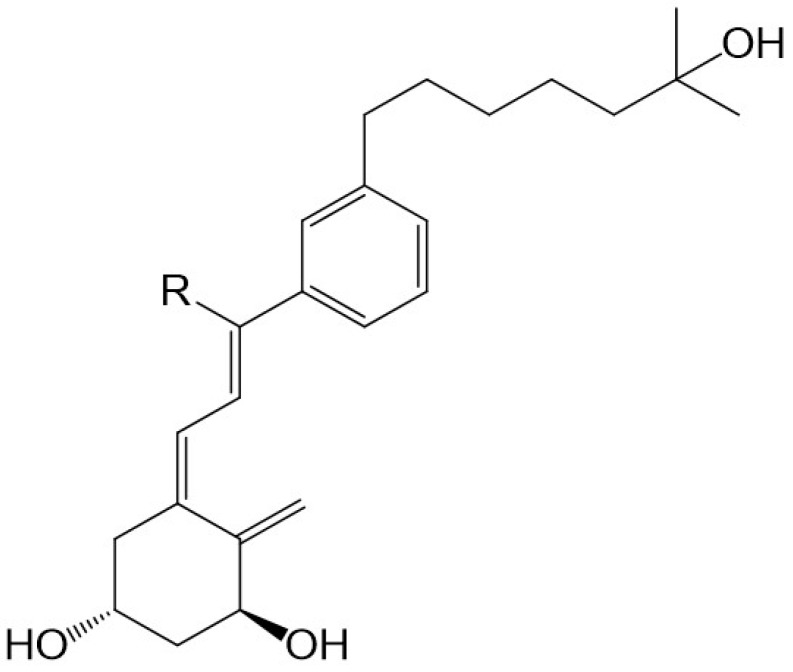
The *des*-C-*m*-phenylene-D-21-*nor*-analogues of 1α,25-dihydroxyvitamin D_3_ (R = Et, *n*-Pr, *n*-Bu, *n*-Hex, *n*-Hept).

There is evidence to support the viewpoint that methyl substituents at C-13 of 13,13-dimethyl-*des*-C,D-1,25-dihydroxy-2-methylene-19-*nor*-vitamin D3 mimic the C-ring of 1,25D3. The 13,13-dimethyl *des*-C,D-analogue of (20*S*)-1α,25-dihydroxy-2-methylene-19-*nor* vitamin D_3_ (Figure 2) still retains much of the activity of vitD [34]. As predicted [29] and confirmed recently [35], even an extensive modification of the CD-ring does not affect the functional activity of vitD as long as the three-dimensional (3D) arrangement of the three 1α, 3, and 25 hydroxyls, responsible for VDR binding, is preserved. As expected, a number of CD-ring modifications resulted in a lowering or complete loss of the undesired calcemic action regarding the development of a potent agent that selectively drives differentiation [30]. A lowering of calcemic activity is also the case for a novel class of five *des*-C analogues with the CD-ring fragment partially replaced by an alkyl chain to mimic the C-ring and an aromatic *m*-phenylene ring replacing the D-ring (Figure 5). Docking studies to the human ligand-binding domain of the VDR led to the design of these analogues. The analogue with an ethyl substituent to mimic the missing C-ring is active against breast cancer cells and, as expected, has negligible calcemic action [36]. These analogues provide important information about the structure–activity relationships regarding CD-ring modifications. This novel class of aromatic-based vitamin D analogues also confirmed that retaining the network of hydrogen bonds of 1,25D3 is crucial for transactivation activity and that aromatic modification of the CD-ring fragment abolishes calcemic activity.

## 7. Future Directions

As described, various modifications to vitD underlie the search for a new drug candidate. Beneficial modifications are those to the A-ring, the triene system, the CD-ring fragment, and the side-chain. An old viewpoint was to make very subtle and single modifications. The current understanding to obtaining the most potent VDR agonist is to combine several modifications at various parts of the molecule. However, the number of possible combinations is numerous. The route to the best combinations remains elusive, because the ultimate outcome of combinations is quite unpredictable. They might result in enhancement or in a complete loss of activity. Even so, several vitamin D super-agonists have been rationally designed and synthesized.

Intuition has mostly driven the design of new vitamin D analogues, coupled with trial and error. A crystal of the full-length native VDR that is suitable for X-ray diffraction study is presently not available. In this case, a classical model [37] that makes use of a truncated VDR protein remains the only available approach to examining the interactions of vitDs with the VDR. This artificial VDR protein has the flexible hinge region cut off to improve the ability of the residual VDR to crystallize. The accumulating of more and more data leads to the viewpoint that the standing model might be of limited significance, because the resulting solid-state structures of various analogues show overlapping of the functional hydroxyls. Very recently, a study of a panel of five analogues of very different structures recorded no detectable differences regarding binding to the VDR [36].

A common understanding is that crystallography plays the major role as the only direct method for studying analogues *per se* and their protein complexes to reveal structure versus activity relationships. Relative spectroscopic methods, such as high frequency nuclear magnetic resonance (900 MHz) [38], are still of a very limited use, as not enough data have been accumulated for a complete signal assignment. Up to now and for complexes of vitDs and the VDR, there is only low-resolution crystallographic data, usually well above 2 Å. We might expect that high-resolution crystallographic data, below 0.5 Å, will give a much better insight into the structural differences between the VDR complexed with various analogues.

However, to make substantial progress and ensure the design of analogues in a truly rational manner an entirely new crystallographic approach is required. It is highly desirable to obtain single crystal high-resolution data of the native VDR, other than for a truncated model protein. Considering the recent developments of crystallography, the time is right to make a concerted effort to obtain the crystal structure of the full-length human VDR *per se* and complexed with vitDs. Automated high-throughput robots now crystallize proteins up to three times larger than the VDR. Additionally, consideration of modern cryo-electron microscopy as a tool for examining a native VDR-retinoid X receptor (RXR) complex is worthwhile. Success in the above directions will open a new era that should reveal the real relationships between the structure and the biological activity of vitDs.

To understand the various biological roles of the VDR there is a need to develop an antagonist of the VDR. The rational design of a potent antagonist is much more difficult than designing an agonist, because there are only few antagonistic structures for use to examine correlations between structure and function. Additionally, computerized docking approaches are especially useful for designing agonists, and the same method is useful to the design of antagonists. The crystal structure of the full-length VDR will be valuable to the design of an antagonist.

## 8. Concluding Remarks

**Figure 6:**
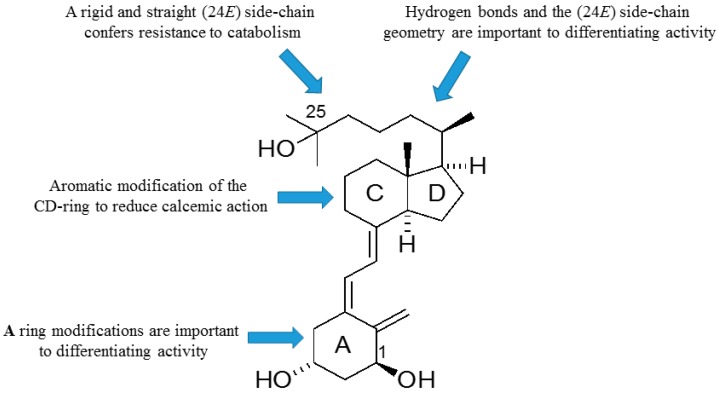
Map of the relationships between structural modifications to 1α,25-dihydroxyvitamin D_2_ and biological actions.

As outlined, the precise nature of modifications to the structure of vitD is important to the gain and loss of particular biological actions. The modified analogues, in turn, are important to developing a better understanding of how vitD can mediate a variety of different biological roles. Of particular interest is the generation of vitDs that are potent differentiating agents and lack calcemic action, for use in studies of cell differentiation and as potential anti-cancer agents. Work over a number of years has generated a range of analogues with very different modifications to the structure of vitD and has correlated their structures with biological actions. A number of important structure–activity rules have emerged (see Figure 6). Analogues with an aromatic modification of the CD-ring fragment have a reduced calcemic action. The hydrogen bonds of the natural 1,25D3 are crucial for transactivation activity, and vitDs with the (24*E*) side-chain geometry combined A-ring modifications are potent anti-proliferative and differentiating agents. Too many structural modifications, a combination of four in the case of PRI-1734, led to a complete loss of biological activity. A rigid and straight (24*E*) geometry of the side-chain confers resistance to catabolism *via* enzymatic hydroxylation by *h*CYP24A1. The provision of a better understanding of how vitD and analogues bind to VDR is also essential to unraveling function. In this regard, the existence of the vitD A-ring in the β-chair conformation, rather than the α-chair conformation, is essential to the differentiating activity of analogues. The application of the rules to activity gained so far should allow analogues to be refined further regarding potency and selectivity.

## Figures and Tables

**Table 1:** Differentiating potency, calcemic action, and binding to the receptor for vitamin D (VDR) of PRI analogues of 1α,25-dihydroxyvitamin D_2_.

Analogue	Differentiation EC_50_ *m* × 10^−11^	Calcaemic Action Ca^2+^ (Serum) *m* × 10^−6^	VDR Binding IC_50_ *m* × 10^−10^
1,25D3	53	107	23
PRI-1907	6	75	62
PRI-5100	113	86	6
PRI-5101	118	90	5
PRI-5201	3	93	11
PRI-5202	2	81	36
